# Managing children with diabetes within the family: Entering into the Diabetes Orbit

**DOI:** 10.1186/s40200-016-0228-8

**Published:** 2016-03-18

**Authors:** Mahnaz Sanjari, Hamid Peyrovi, Neda Mehrdad

**Affiliations:** 1 School of Nursing and Midwifery, Tehran University of Medical Sciences, Tehran, Iran; 2 Endocrinology and Metabolism Research Center, Endocrinology and Metabolism Clinical Sciences Institute, Tehran University of Medical Sciences, Tehran, Iran; 3 Nursing Care Research Center, Department of Critical Care Nursing, School of Nursing and Midwifery, Iran University of Medical Sciences, Tehran, Iran; 4 Diabetes Research Center, Endocrinology and Metabolism Clinical Sciences Institute, Tehran University of Medical Sciences, Tehran, Iran; 5 Elderly Health Research Center, Endocrinology and Metabolism Population Sciences Institute, Tehran University of Medical Sciences, Tehran, Iran

**Keywords:** Family, Managing of children with diabetes, “entering into the orbit of diabetes”

## Abstract

**Background:**

Diabetes is the disease of family and parents of children with diabetes face different problems which concerns meeting the developmental needs of children and daily control of children with diabetes. This article aims to explain how to manage diabetes around the child’s life within the family.

**Methods:**

In this qualitative study, data was collected through semi-structured interview technique and was analyzed using Grounded Theory approach. The process of data collection was carried out by purposeful sampling. The participants included 13 individuals from nine families (11 parents and two children with diabetes). The research environment was health centers in Iran providing care to the families of children with diabetes. Data analysis was performed using Corbin and Strauss approach. Data was analyzed with using MAXQDA software (version 10).

**Results:**

The core category of “Entering into the Orbit of Diabetes” addresses the story of how to keep track of managing children with diabetes within the family which included Main categories “bitter taste of sugar”, “drawing coordinates of diabetes”, and “taking control of diabetes”.

**Conclusion:**

The outcome of “enter into the orbit of diabetes” results capturing the control of diabetes. The findings of the present study may play an integral part to help households with practicing appropriate strategies for the management of children with diabetes.

## Background

As a chronic disease, diabetes is the illness of all family members [1, 2]. Diabetes as a “grieving diagnosis” conquests the various life aspects of families of children with diabetes [3–5], It means, the whole process of disease is followed by a series of complications and decisions makings which involves all members of the family, friends, and health care providers are involved [6]. Due to the importance of family interactions in the control of Type 1 Diabetes, it is attributed as the “Family Disease” [7].

Diabetes is one of the few chronic diseases which requires care and attention in the course of daily living activities [1]. The issues concerning families of children with diabetes are not only glucose monitoring, nutrition and physical activity [8], but also is the daily control of the disease inside and outside the home [9]. Families’ roles, particularly when children suffer from chronic illnesses are changed [10–12]. The family shapes the context of patients so that in the process of providing care, parents play a key role in focusing on the individual nature of the children [6]. The study showed that for families of children with diabetes, the “child” has higher priority than the “diabetes” [13].

Apparently, the diabetes also comes with some challenges. Several studies have paid to these challenges such as parental worries, self-care, as well as psychosocial problems of children and adolescents with diabetes [14–16]. Given the increasing outbreaks of diabetes among children and adolescents and the challenges of caring for this age group on one hand [17] and the necessity of family members’ contribution to the management of diabetes on the other hand [18–20], many more studies are needed to be carried out on the process of care concerning cultural, social, economic, and family characteristics of households to render effective diabetes management [21, 22].

Although several clinical guidelines and protocols have been developed for the control of diabetes, but managing children with diabetes is somehow a different issue, regardless of the guidance, which occurs in the family. At this point, it is necessary to know how the family, especially in the early stages of the diabetes diagnosis, tries to manage its concerns. This multidimensional process can be traced the most from the perspective of those who have experienced it. This study is part of the PhD thesis in which the process of managing children with diabetes in the family was investigated by the Grounded Theory.

## Methods

### Study design

According to the aim of the study, which explains how to manage children with diabetes in the family, a naturalistic approach was selected to explain the unknown aspects of this process in the Iranian family. Accordingly, the participants’ experiences were analyzed using Grounded Theory approach of Corbin and Strauss to study the underlying process in the context [23].

### Participants

Participants in this study were families (father, mother, sister, brother) of children with T1DM diabetes and also the children with diabetes who lived with their parents. Those participants willing to participate in the study were required to have the ability to understand and speak Farsi. In the children with diabetes, diagnosis must be defined at least more than one year and without any other chronic diseases.

### Sampling

The participants were selected purposefully according to the grounded theory approach. After analyzing the first interview, the subsequent participants were selected based on the purposeful sampling. Thereupon, theoretical sampling was used in order to extent of development and the enrichment of categories and their dimensions. The research population was families of children with diabetes who were referred to the diabetes clinics, NGOs and societies in Tehran province.

Based on data analysis, the researcher tried to consider families of children with diabetes who differed in terms of age, gender, education, economic status, size as well as the child's age, sex and birth order.

Upon receiving the approval of Ethics Committee, the researcher referred to the above mentioned sites and selected eligible participants. The researcher explained participants the purpose of their research and written consent were obtained confessing the participants’ willingness to contribute in the study. In the case of young children, the informed consent was signed by both family and offspring.

### Methods of data collection

In order to collect demographic information a form was used including age, sex, education, marital status, economic status and size of the family. This form also included age, gender, duration of diagnosed diabetes, and the birth order of the children with diabetes. The semi-structured interview was used to explain the process of managing children with diabetes within the family. In the process of data collection, nine family were interviewed encompass in form of family and individual interviews (13 participants) including 11 parents and also two children with diabetes in the time duration of 40–100 min with the mean of 69 min. All the interviews were recorded upon the permission of the participants. The open-ended and semi-structured questions asked during the interview were focused on how to manage children with diabetes within the family. Data obtained during each interview determined questions of the subsequent interviews. The samples of questions are presented in Table 1.

**Table 1:** Selected interview questions

Main questions	What did happen to you following the diagnosis?
	How do you manage children in daily routine?
	What are the obstacles of controlling children with diabetes?
	Which resources did you use to overcome the obstacles?
	What changes should be done to control children with diabetes?
Probe questions	What was your reaction in those circumstances?
	Please give us an example?
	What is effective to take control of the situation according to your experiences?

### Data analysis

Corbin and Strauss approach (2008) was used for the analysis purposes in this study [23]. The management of data analysis was through using MAXQDA software version 10. From the initial stages the data was assessed on a word by word and line by line basis. At this stage, researcher listened to the interview several times, and then transcribed the audio into the text, and then analysis was followed in micro and macro level. Different methods of qualitative analysis, such as “Use of questioning”, “Looking at emotion”, “Making comparison”, as well as “Using the flip-flop technique” were used in micro and macro level analysis. For example the subcategories of “Defining of diabetes “, “Diabetes rush “and” Sticking to a behind the window diet “was developed by using the “flip-flop technique” and “Looking at emotion technique” respectively. Also the context based categories such as “Leakage of money out of diabetes split” and “Perception of diabetes by others” was grown by “Paradigm Method” and drawing “Matrix”. In order to integrate the categories, the researcher specified the core category by mean of making use of writing Memo, drawing diagram and deep immersion into the data.

### Trustworthiness

In order to fit the data with the research findings, the researchers allowed the participants’ experiences and interpretations to direct the study in a way that the research findings are in line with the real-life experiences of families of children with diabetes. The researchers did their best to provide the concepts in a tangible and understandable way to make families of children with diabetes able to understand them. At the end of each interview, participants were also asked to approve the concepts obtained. Since this study is the first experience of conducting a qualitative study for the researcher, she did her best to observe the required sensitivity needed for achieving and discovering the concepts through spending sufficient time and multiple reviewing of data.

### Ethical considerations

The researcher explained all probable benefits or damages to the families, and written processed informed consent was obtained from the participants. Also, the research finding was reported without disclosing the identity of study participants. This could claim reserving the rights of all individuals involved in this study.

This study approved by the ethical committee of Tehran University of Medical Sciences with approval number of 3548/130/ D/9.

## Results

In the analysis, 200 open codes in 12 sub categories, three main categories, and one core category were used to describe the process of managing children with diabetes in the family. Once in practice, the average age of children with diabetes and the mean duration of diabetes were reported 11 years and 5.5 years, respectively. As well, the minimum and maximum duration of diagnosed diabetes were two and 13 years, respectively. The core concept and main categories demonstrate in Table 2.

**Table 2:** The core concepts of “Entering into Diabetes Orbit”

Main categories	Sub categories
Bitter taste of diabetes	Diabetes rush
	Sticking to a behind the window diet
	Leakage of money out of diabetes split
	Diabetes image in the mirror of others
Drawing the coordinates of diabetes	Defining of diabetes
	Dealing with the consequences of diabetes
	Confronting with caring obstacles
	Keep diabetes hidden
Taking control of diabetes	Being equipped with knowledge and expertise
	Grabbing the rein of diabetes
	Living with the limitations
	Founding the order at home

### Bitter taste of diabetes

This category includes four sub-categories including, “Diabetes rush”,” Sticking to a behind the window diet”, “Leakage of money out of diabetes split”, and “Perception of diabetes by others”. Coincident with confronting the sad feeling of the diagnosis, the family finds itself facing with the challenges caused by diabetes. The “bitter taste of diabetes” is felt whether at the early stages of disease, at the time of detection of diagnosis, or during the hospitalization, and even while returning home.

One of the most important challenges the family will be facing is the food restrictions imposed on children with diabetes by compulsion which are accompanied by emotional and care giving tensions. One must also consider that diabetes is a costly disease, especially if be coupled with lack of knowledge of proper treatment which can result in wasting most of the household income. The judgments of others and the reactions of people toward the disease will undermine the growing bitter sense of diabetes. The consequence of this category is the overwhelming feeling of tension and discomfort, disruption of the normal process of life, and the need for further understanding of diabetes.

#### Diabetes rush

Diabetes rush is the initial point in which the family comes to terms with the challenges of diabetes. Family members spending their normal life are now confronting an incursion of diabetes. By this time, family acts as an atom circuiting in a certain orbit. So, suddenly hit by a diagnosis brings an awful lot to consider for the whole family. It is worth knowing that the diagnosis is not a change initiated gradually. In the context of Iranian families, “Diabetes rush” includes shock, lack of awareness of diabetes and its subsequent behaviors, difficulties of hospitalization, as well as incurable nature of diabetes.

Stress and anxiety following the diagnosis are a natural reaction experienced by all families*.”.... I was shocked when I heard about her diabetes. It was very difficult* (3-mother). Hospitalization following diagnosis is another distress that parents find it difficult. “*… when her physician told us she must be hospitalized as soon as possible, this stoke on my head like a sledgehammer…”* [2].

#### Sticking to a behind the window diet

Dietary restriction necessitated by diabetes is one of the issues that are accompanied by psychological and care challenges for parents of children with diabetes. The usual positive attitudes of parents towards the child’s needs and wants is now highly affected by modifications that must be applied to control the disease. This may rather bring a further bitter taste for the family. The results of this category reveal that these challenges get more pronounced for a family who is facing the continuous demands of child for which there exist no other way than restriction.

The second participant describes her first encounter with these limitations in the hospital as follows,*“…. the nurse told us your child should only eat this much rice, and the chicken that I put for her, nothing more! I told her let me give her a little porridge, and she strictly said No!!!* [2]*”*. Dealing with such a food restriction makes family to get away from their normal life because they do not have enough knowledge and skills about how to carefully manage their child's condition as part of daily life. “*The year when she was diagnosed with diabetes one month later was her birthday which was a tragedy for me. How we were supposed to arrange a birthday for her when she couldn’t even eat two spoons of porridge* [2]*.*

#### Leakage of money out of diabetes split

The cost implication of diabetes is referred as another issue contributing to the bitterness of diabetes. Although participants were selected from different economic categories, but funding for treatment is a challenge for all families. Diabetes, as a split in the family wallet vacates the family resources very fast and obviously insufficient family income may worsen the situation.

Such a challenge is more highlighted when the family lacks the essential skills necessary for controlling diabetes effectively.”… *You see the costs are very high, every single test costs me about 20 cents, and each syringe is about 4 dollars. She needs 4–5 injections per day* [7]*.* Apart from the high cost of treatment that is added to the cost of living, poor economic conditions of the family can also delay managing the condition of a child with diabetes. As a participants said, *“his physician told us why we take him so late for visit, I was driven crazy. You see I have two children with diabetes and each need to be tested. The device and the strip are expensive how I should afford this much cost by having three children” (9-mother).*

#### Perception of diabetes by others

Mostly, people in their daily life pay attention to their understanding of how other perceive them. Taking this to account, for a family who has just confronted with the storm of diabetes and is involved with the troubles caused by the disease, it seems to be more grappled by the issue. This is due to the fact that shaping such conception is generally surrounded by the judgment and pity of others.

*“…, my daughter came to home one day as white as a sheet and told me that she had a struggle at school that day. She had been* teased and taunted *by her classmates as diabetics!!* [6]*”.* Expressing too much sympathy and pity to children with diabetes mostly bring discomfort or their families.” *Behavior of others towards my kid is simply destructive and I can't stand such a behavior* [2]*.* Due to the cultural characteristics of Iranian families, mothers have been usually ruled against by judges. As one mother said, “my *in-law families think that I’m responsible for such a situation. Their blames fall on me for the poor nutrition of my kids! And my son also holds a grudge against us* [1]*”.*

### Drawing the coordinates of diabetes

The family tries to form a new concept of life which is living with diabetes through drawing the coordinates of diabetes. This is mainly due to the fact that the family demands to form an understanding of the current situation. This makes them able to provide an appalling insight into the disease and then manage the child with diabetes. Accordingly, family, emotionally affected by the diagnosis, tries to form a meaning of diabetes, meanwhile get familiar with the consequences of diagnosis, particularly its responsibility in providing care. This care may vary during the life span of the child but is never stopped. For the same reason, diabetes should be clearly defined for the family before anything else to make them able to design and implement the care plan.

This category covers four sub-categories including, “defining of diabetes”, “dealing with the consequences of diabetes”, “confronting with caring obstacles “, and “keep diabetes hidden “.

#### Defining of diabetes

The majority of families find a better understanding of the situation through defining the diabetes. This definition alleviates the deep upsetting of diabetes imposed on the family life. The majority of families complain about the annoying effects of diabetes. This implicitly shows the deep suffer confronted by the patients subsequent the diagnosis.

One of the participants described diabetes as a misery and expressed,” *I was shocked when they suddenly hospitalized him, tomorrow, then I found what a misery has happened to me! (3-father)” .* Despite all the difficulties associated with diabetes, some, however, find it as having positive implications too. “*In my idea as adults are satisfied through passion the same happens to children through eating. So, when she learns to say “No” to herself, she is getting matured from the very beginning!* [2]*”.*

#### Dealing with the consequences of diabetes

Through defining diabetes, the family considers all the implications of diabetes in its daily life, and obtains the necessary incentives which arm it against the probable consequences of diabetes. The families of children with diabetes suffer from a wide range of concerns, including the constant existence of needle in their everyday life, persistence fear of hypoglycemia, fear of complications, as well as the panic of diabetes development in other children.

The horror of sudden hypoglycemia is one of the most important issues faced by families at all stages of handling children with diabetes*“… what the most terrifies me is sudden sugar drop…. I can’t get a wink of sleep since all I think now a days is that what if I find him in coma… I can’t stop the very thought of losing him for dropping his blood sugar while sleeping* [4]. The other consequence making the family constantly think of is the other children's chances of developing diabetes. *“When my older daughter was diagnosed with diabetes we were always afraid of happening the same for our other kids. The same happened and my second kid became diabetic 1.5 years later (father-8)”*, said a participating father with two children with diabetes.

#### Confronting with caring obstacles

Given to the chronic nature of diabetes, the family is punched down to a context of continuous care. The care obstacles are one of the reasons making family to draw the coordinates of diabetes in the context of its constant effort to find a better understanding of disease. Facing with the task of care, the family pays attention to what hinders providing the best possible care.

One notion excessively referred to by parents is the lack of awareness about the principles of diabetes control. “*…. I was touch-and-go for a while; I didn’t know what to do… (3-mother)”.* Lack of support provided by other members within the family is also an important notion pointed out by mothers. *“… Diabetes needs companionship, but I play a lone hand. His father is busy with his jobs and when he comes home at nights he thinks this is none of his business* [7]*”.*

#### Keep diabetes hidden

From the very beginning of diagnosis, the family finds the diabetes as an inseparable part of family life and the individual child. Taking this into account, the family seeks to further increase its knowledge of the disease. This further knowledge doesn’t merely include searching the existing literature on diabetes; in turns the family tries to discover the implication of diabetes in terms of its own setting.

One reason to do so is the surrounding misinterpretations and improper judgments towards the disease. Hiding the diagnosis, even for a short period of time, is a way to protect the family against the reaction of others*.”….we didn’t let on the family…,it tears my hair out talking to them. I know they don’t mean it but sometimes I lose my temper!”* [9]*.*

### Take control of care

The family comes to the conclusion that it requires to be armed with awareness, knowledge and skill while experiencing the “bitter taste of diabetes” and “dealing with the care obstacles”. At this point, as a result of the challenges and problems brought by diabetes the family finds itself losing the track of its life. Therefore, there seems to be a need to get more involved in taking the control of its own life. This category entails four sub-categories including, “being equipped with knowledge and skill”, “living with limitations”, “grab the rein of diabetes”, and “ruling order at home”.

#### Being equipped with knowledge and expertise

One of the primary approaches taken into account by family to provide care is being fully equipped with knowledge and experiences. This is mainly due to fact that, lack of sufficient knowledge is one of the initial obstacles with which the family is confronted with.

As an initial step, the educational needs are responded by health care providers at the hospitals. To seek the care demands, the parents insist to learn in spite of being shocked and fatigue. “…*we kept in picture of care giving at the hospital although we were not in a good mood. They told us you should learn how to inject insulin and to check the blood sugar, and then we let you go* [4]*”.* A widely used resource was the care-based educational classes provided by some institutions. “*… I got familiar with a peer in the same ward, her daughter also had diabetes, she introduced the Gabric NGO to me, my son and I both took part in the classes, they were practical…* [1]*”.*

#### Living with the limitations

Although the family paves the way to acquire knowledge and expertise, but it doesn’t guarantee that family moves in the direction of providing the best of diabetic care and management. For this reason, the family prefers to use more restricting approaches in order to supply itself with more opportunity to improve his abilities and brings more assurance to provide care. In opposite with the sub-category of “sticking to a behind the window diet” which is confronted by parents out of their willing, during this phase the family is reluctantly setting the limitations during this phase to take a better control of diabetes.

Resistance towards the demands of the child is of the most accessible approaches used by parents. “… *When she insists on eating something, I don’t let her …but you know it hurts to do so every day* [7]*“.* Such limitations, however, are not only set for eating but at the early stages of diagnosis when the family seeking to focus more on the situation tries to limit social interactions as well*.”…. when we gathered around, they forgot my daughter has diabetes and brought sweets. They couldn’t figure out how hard it is…. Then so I preferred to put our relationship on a downward spiral (8-mother)”.*

#### Grabbing the rein of diabetes

The findings have revealed that at the early stages of diagnosis the family has not yet fully learnt how to move along with the diabetes. Therefore the family tires to take wise and purposeful steps towards a better diabetes care. The parents now find themselves as being responsible toward the diabetes management and do their best to optimize the situation in a way to grab the rein of diabetes. In the course of taking the control of diabetes, parents are fully concentrated on managing the disease; therefore they avoid threatening their child's health. Grabbing the reign of diabetes encompasses some changes and steps taken by family to take a better control of diabetes. This may include a wide range including, accepting the diagnosis, preparing the family setting, leaving job, encountering previous fairs, as well as impatiently trying to take control of diabetes.

The management of children with diabetes is a critical issue for the family to the extent that they take action upon even prior to fully accepting the disease. However, the family comes to the term that they need to accept disease to afford a better control of diabetes *“…I should stand by the situation, I don’t have to settles and all I should do is to control my child's blood sugar. I buy it that my stress will only set my husband and children off. I’m doing the best of me to keep my child healthy….(mother- 3)”,“You know risk of not controlling was quite large, the problem was that 20 % of his intelligence was going to fade. I wouldn’t like to walk on eggs* [2]”.

#### Founding the order at home

In quest of a better control of diabetes, the family swiftly understands that one of the requirements to grab the rein of diabetes is founding the orders and disciplines within the family. The necessity to establish such discipline is mainly rooted in the continuous need for regular care of children with diabetes.

“… *A diabetic person can’t be let it go… they need to get every single thing scheduled…* [7]*”.* Participant eight sees this order even outside of the house as she says *“I told them this snack is for your first break, and that is for the second break….otherwise their blood sugar had a sudden raise. All pick on me for this but don’t care I just care their health (8-mother)”*

## Discussion

**Fig. 1:**
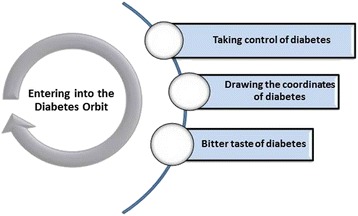
The process of managing children with diabetes within the family

The results of data analysis have shown that entering into the orbit of diabetes represents the process of managing children with diabetes within the family (Fig. 1). The nature of diabetes requires the family to confront with the tensions of diagnosis even while involved with the responsibility of taking care. The personality of a child with diabetes is formed within the context of the family. At the early diagnosis of diabetes the family is much concerned about the diabetes rather than the individual child, and finds the disease as part of the child. For this reason, the family does its best to grab the rein of diabetes and control his child through managing the disease to return the piece to the family.

At the start of entering into the orbit of diabetes, the family initially conceptualizes the bitterness and dangers associated with diabetes. Subsequently, the family tries then to define the care to obtain a better understanding of the disease and to draw the coordinates of diabetes. In the following, family concerning on the disease does his best to prevail the disease.

A significant degree of tension and concern is experienced by the family of children with diabetes during the initial phase of managing the diabetes. Several other studies have also revealed such a “bitter taste” for families. The result of the study on children with T1DM showed that being diagnosed as having diabetes will bring a wide range of problems not only for the child but also for all family members. So that the whole family aspects will be subsequently affected by the problems caused by diabetes [8].

Diabetes rush brings worries and anxiety for the family. A meta-analysis on studies of the family with children with diabetes was indicative of anxiety and tension among families. This study was suggestive in terms that the practice of food prohibition for children is a tension causing stimulus confronted by family at the early presentation of disease [24].

Part of this prohibition is, however, the act of health care providers for a better management of disease. Yet, this also comes from the lack of awareness of the families about how to provide the child with an appropriate diet. A qualitative study carried out on Iranian families with children with diabetes has published similar findings which may be due to the similar social and cultural context with that of our study. In this study worries about social issues including inappropriate social reactions and negative attitude of relatives toward a child with diabetes have been introduced as core concepts [16].

Another source of tension within the families is the high-cost therapy of disease. One point highlighted in our study is the high cost of treatment. This is not, however, considered as a problem and issue in developed countries. Meanwhile, financial problems associated with medication costs appear as an initial category in our study.

What explains these different findings may rely on different type of health care systems and insurance coverage in any country. Iranian families having children with T1DM are not adequately covered by supportive systems. For the time being, apart from a few non-governmental organizations which are more involved in pay-in-turn educating services to patients and their families, there exists no coherent institution for financial support of Iranian families who have children with diabetes.

In addition, the amounts of supplemental insurance paid for both acute and chronic diseases in Iran are the same. This makes funding for diabetes as a chronic disease which requires contentious care for families. The result of one study was indicative of the high cost of treatment for each person with diabetes. This amount, however, varies according to gross national income and the economic level of each country [16]. Drawing the coordinates of diabetes which occurs following confronting diabetes consequences and care obstacles is taken into account as the next phase of the process of managing children with diabetes. The results of a qualitative study emphasized on the higher responsibilities of parents of children with diabetes due to the additional task of caring. Accordingly, “Intensive daily routine” was a concept concluded in this study [8].

As the results suggested, the inability of parents to control the disease was one of the consequences of diabetes. The result of one study showed that a child’s suffering from diabetes is one of the worst experiences of families, because parents may feel that their ability to protect their child against physical and psychological injuries is reduced [8]. The non-participation of other family members in the care, especially fathers, is noted as another care barrier in this study. The traditional culture of Iranian families, fathers in a way that, they are mostly recognized as breadwinners. Therefore, their presence and participation in care may reduce when they try to earn much more money to tackle the high cost of treatment. This is also noticeable in other culture in which fathers are not strongly involved in the first contact care of their children with diabetes [25]. However, the result of one content analysis study revealed that fathers of children with diabetes are actively contributing to provide daily care to their children [13].

The results raised by the empirical studies of Iranian families showed that families initially focus on the mere disease. This is mainly because in diabetes, opposite to other diseases the highest portion of care is assigned to the family. Considering the fact that there is not any supportive systems like social health workers to follow the living conditions of such families, thence families have to deal themselves with their care responsibilities without being socially supported. In such a situation families seek to strategies such as “defining diabetes” and “keep diabetes hidden” through which they find more opportunity to shape diabetes in their mind. Moreover, the family finds sufficient time for a proper control of disease through limiting the scope of social interactions and hiding diabetes diagnosis. Through limiting the scope of social relations provide more opportunities to control their disease. Similar to those of our study, one study revealed that ordinary people know a little about diabetes. For the same reason they are reluctant to reveal the disease outside of the family since they feel annoyed by the reactions and FAQ of others [8].

The complementary part of entering into diabetes orbit is taking control of care. At this phase, acquiring knowledge and expertise, taking control of diabetes, and setting restrictions as well as the establishing order in the family are strategies aimed at shaping the management process. In fact, at this stage families find that diabetes is a component that is added to all aspects of their life. Strategies used by the family during this phase reveal that the family is initiating to build the cornerstone of a new family in which diabetes is one of the main embedded components. The time lapse subsequent to diabetes diagnosis allows the family to discover that diabetes is deeply rooted in the everyday life of the family. Other studies also showed that the skill acquired by the knowledge and expertise is another strategy used by families to control diabetes [8].

The core concept of “entering into diabetes orbit” encompasses a spectrum in which the family is initially involved in diabetes but is gradually encouraged in some activities through drawing the coordinates of diabetes. At the end of this process, the parents take control of diabetes. The core concept proposed in one study, conducted on fathers with children suffering from diabetes, was described as “from sadness to action”. This is mainly because fathers studied in this survey were recognized as being firstly shocked by diagnosis and then engaged actively to take part in the care, and got involved in the daily care of children with diabetes [13].

## Conclusion

“Entering into the orbit of diabetes” implies a journey of family to another orbit in which the whole family life is highly influenced. Followed by diagnosis and experiencing the subsequent bitter taste of disease, the family soon finds itself as being distinct from other ordinary families and come to the conclusion that the family life is permanently changed. In order to respond to such a change and maintaining the life stream, the family is forced to such an obligatory journey.

In the process of managing a child with diabetes, the family initially has a passive role and is negatively affected by the situation. This is similar to what happened at the early stage of disease diagnosis in which the family tries to explore the coordinates of diabetes to obtain a better understanding of disease. Likewise, the family tries to be more actively engaged in the process of controlling the child with diabetes through which enters into the diabetes orbit. The outcome of “entering into diabetes orbit” is that the family obtains a relative understanding of disease and learns about the weak and strong point of care giving. Accordingly, the family takes are recognized and the needs are assessed. This paves the way for family to equip itself to knowledge and skills meanwhile to initiate care giving to the child with diabetes.

The findings of this study can help the families to develop proper strategies to form the process of controlling children with diabetes within the context of family. These findings may be also practical to be used by health care providers in order to design suitable protective and also educational systems particularly at the early stages of confronting with diagnosis by the families.

### Study limitations

Of the limitation of the current study is the subjective nature of the study which makes the ideas and beliefs of the researcher to affect the process of data analysis. In fact, ignoring or putting a side such bias is not fully possible particularly because the process of managing children with diabetes within the context of Iranian families still remains as a critical issue for the researcher.

To remove such a limitation, the researcher tried her best to identify all her presumptions and intellectual beliefs prior to the analysis to eliminate the probable bias. Besides, the researcher was well aware of these beliefs throughout the research and tried to control them by means of multiple reviews succeeding to data collection as well as data analysis. In addition, this study is conducted on those families referring to the diabetes clinic or other specialty health care centers of Tehran University of Medical Sciences which limited data variability and transferability. To compensate part of these limitations, the researcher conducted interviews with families from different social classes.
